# Pharmaceutical Equivalence of Clarithromycin Oral Dosage Forms Marketed in Nairobi County, Kenya

**DOI:** 10.3390/scipharm85020020

**Published:** 2017-04-25

**Authors:** Rebecca O. Manani, Kennedy O. Abuga, Hezekiah K. Chepkwony

**Affiliations:** 1 Department of Pharmaceutical Chemistry, School of Pharmacy, University of Nairobi, P.O. Box 19676, Nairobi 00202, Kenya; koabuga@uonbi.ac.ke; 2 National Quality Control Laboratory, P.O. Box 29726, Nairobi 00202, Kenya; kcheph@yahoo.com

**Keywords:** clarithromycin, comparative dissolution, similarity factor, pharmaceutical equivalence, quality

## Abstract

Clarithromycin is a broad-spectrum semi-synthetic macrolide indicated for treatment of pneumonias, *Helicobacter pylori*, and chlamydial and skin infections. The object of this study was to evaluate the pharmaceutical equivalence of 14 generic clarithromycin products marketed in Nairobi County, Kenya, to the innovator products, using in vitro dissolution profiles and similarity factors (f_2_). Further, dissolution profiles of four innovator formulations manufactured in different sites were compared. Fourteen clarithromycin tablets/capsules and four suspensions were subjected to assay and comparative dissolution runs at pH 1.2, 4.5 and 6.8, for 60 and 90 min, respectively. All products complied with pharmacopoeial assay specifications. However, significant differences were observed in their dissolution profiles. The non-compliance rates for tablets/capsules were 50% at pH 1.2, 33% at pH 4.5 and 50% at pH 6.8, while none of the four suspensions were compliant. Overall, only four (25%) products complied with the specifications for similarity factor. The results obtained indicate that a significant percentage of generic clarithromycin products are pharmaceutically non-equivalent to the innovator products, and that assay and single-point dissolution tests are insufficient demonstration of equivalence between the generic and innovator products.

## 1. Introduction

Clarithromycin is a broad-spectrum semi-synthetic derivative of erythromycin with activity against a wide range of Gram-positive and Gram-negative bacteria, as well as the atypical microorganisms *Mycoplasma pneumoniae*, *Chlamydia trachomatis*, *Chlamydophila pneumoniae*, *Legionella* spp., *Borrelia* spp., *Mycobacteria*, *Ureaplasma*, and *Toxoplasma*. It is indicated in the management of atypical community acquired pneumonia, chlamydial infections, legionella pneumonia, and acute non-specific urethritis, as well as skin infections, disseminated mycobacterial infections, and the eradication of *Helicobacter pylori* as a component of triple therapy in peptic ulcer disease [1,2,3]. It has higher oral bioavailability and fewer gastrointestinal side effects than erythromycin [1,4].

**Figure 1:**
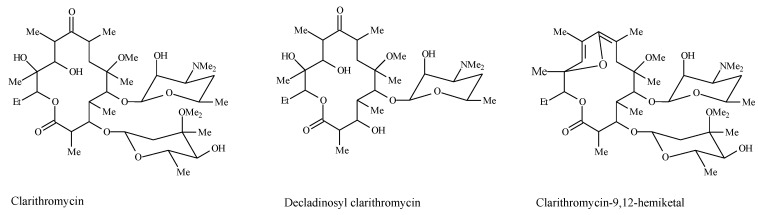
Chemical structures of clarithromycin and degradation products.

Clarithromycin has poor aqueous and pH-dependent solubility with dissolution rate-limited absorption corresponding to Biopharmaceutics Classification System (BCS) Class II [3,5,6,7]. The drug undergoes rapid degradation in strong acid to form decladinosyl clarithromycin and clarithromycin 9,12-hemiketal whose structures are shown in Figure 1 [6,8].

Previous studies have reported that clarithromycin undergoes rapid degradation under conditions of low pH that exist in gastric fluid [6,9]. One such study reports 25% drug degradation at pH 2.0 within 30 min of incubation, while incubation at pH 1.5 yields 70% drug degradation within the same period [9]. Given that the gastric and intestinal residence times of clarithromycin are 0.5–2 h and 3–6 h, respectively, and since its systemic bioavailability is dependent on gastric stability and intestinal absorption [10], protection from acid degradation may play a key role in enhancing its bioavailability. Formulation approaches aimed at stabilization of clarithromycin under acidic conditions have included enteric coating, use of bioadhesive and mucoadhesive polymers, as well as amorphous solid dispersion matrices [5,6,10]. Clarithromycin is an essential antimicrobial agent, featured in the WHO model list of essential medicines for use in combination regimens for eradication of *H. pylori* in adults [11]. It is also a component of drug combinations used in the treatment of mycobacterium avium complex (MAC) in HIV-AIDS patients [12,13]. Quality problems associated with generic products of clarithromycin have been reported in the past, with a significant percentage of generic products failing to meet pharmacopoeial specifications for assay and dissolution, as well as failing the test for equivalence to innovator products [14,15,16].

In Kenya, the Pharmacy and Poisons Board (PPB) is the regulatory body responsible for approvals, granting of market authorization of drugs as well as post market surveillance. Previous market surveillance endeavors by the PPB have focused on antimalarial, anti-tubercular, and antiretroviral drugs, leaving out other categories of medicines, notably antibiotics including clarithromycin. As at March 2014, there were about 49 brands of clarithromycin oral formulations registered by the PPB in Kenya [17]. There are no published reports of comparative dissolution or bioequivalence studies on these generic clarithromycin oral formulations in the Kenyan market. Given that clarithromycin belongs to BCS Class II, comparative dissolution studies are necessary to establish the pharmaceutical equivalence of generic formulations with the innovator products, Klacid^®^ tablets, and suspension (Abbott Laboratories Ltd., Johannesburg, South Africa), which is an important pointer to their clinical efficacy.

The aim of this study was to evaluate the pharmaceutical equivalence of generic clarithromycin products in comparison with innovator products by Klacid^®^ (Abbott Laboratories Ltd., Johannesburg, South Africa) through comparison of their dissolution profiles.

## 2. Materials and Methods

### 2.1. Samples

Clarithromycin samples were obtained from selected private retail pharmacy outlets within Nairobi County, Kenya. Clarithromycin suspensions (125 mg/5 mL) and clarithromycin (500 mg) tablets/capsules were sampled for analysis. One batch each of the available products was obtained through purposive sampling over a period of three months. The innovator comparator products, Klacid^®^ tablets and suspension (Abbott Laboratories Ltd., Johannesburg, South Africa), were similarly obtained. Additionally, one batch of Klaricid^®^ tablets and one batch of suspension (Aesica Queenborough Ltd., Kent, UK) were obtained from Bordon Hampshire, England, for comparison purposes.

### 2.2. HPLC System

Experiments were carried out using a Shimadzu Prominence high performance liquid chromatography (HPLC) system (Shimadzu Corp., Kyoto, Japan) comprising a CBM-20A Prominence communications bus module, an SPD-M20A Prominence UV/Visible photo diode array detector, an LC-20AD Prominence solvent delivery system, and an SIL-20AC Prominence auto sampler. The temperature was controlled using a CTO-20AC Prominence column oven with a block heating type thermostatic chamber, while the liquid chromatography (LC) system was controlled by LCSolutions Software Ver. 1.22, SP1. A Waters XTerra RP18, 5 µm, 250 × 4.6 mm ID chromatography column (Waters Corp., Wexford, Ireland) was used as the stationary phase.

### 2.3. HPLC Method

A published stability-indicating HPLC method for separation of clarithromycin and related substances was applied [8]. The method was verified before application for sample analysis. The optimum chromatographic conditions were established as a mobile phase consisting of acetonitrile, 0.2 M phosphate buffer (pH 6.8), and water (40:3.5:56.5, *v/v/v*) delivered at a flow rate of 1.5 mL/min through an XTerra RP C_18_, 5 µm (250 × 4.6 mm ID) column maintained at 56 °C and UV detection at 205 nm.

Clarithromycin working standard (96.7% *w/w*) was used for determination of clarithromycin content in the assay and dissolution tests. Clarithromycin standard solutions were prepared using acetonitrile-mobile phase (40:60) to a concentration of 1 mg/mL. Decladinosyl clarithromycin content was determined using relative retention time (RRT) reported in the literature [8] because the working standard was not available. During quantification of the decladinosyl clarithromycin content, normalization factors reported in the literature [8] were used.

### 2.4. Assay

#### 2.4.1. Tablets/Capsules

Pooled powder from 20 tablets/capsules was weighed in triplicate whereof the samples were dissolved in acetonitrile with the aid of mechanical shaking for 45 min to a concentration of 2 mg/mL. The stock solutions were diluted with mobile phase to a final concentration of 1 mg/mL, filtered through 0.45 µm filters, and subjected to HPLC analysis.

#### 2.4.2. Suspensions

Clarithromycin granules for suspension were reconstituted using purified water according to label instructions. The contents of three bottles were mixed in a beaker and clarithromycin extracted sequentially using 0.067 M dibasic potassium phosphate and methanol for 30 min each as prescribed in the United States Pharmacopeia (USP) [18]. Dilutions of the resulting solution were made using mobile phase to a concentration of 1 mg/mL and filtered through 0.45 µm filters before HPLC analysis.

### 2.5. Dissolution Profiles

Samples were subjected to dissolution profile tests on a Labindia DS 8000 dissolution tester (Labindia Analytical Instruments Pvt. Ltd., Maharashtra, India) equipped with USP Apparatus 2, 900 mL of dissolution media, and a bowl temperature of 37 °C, and stirred at 50 rpm. The dissolution media used were 0.1 M HCl (pH 1.2), 0.1 M acetate buffer (pH 4.5), and 0.2 M phosphate buffer (pH 6.8), all prepared as per USP specifications [18]. Six tablets/capsules and 6 × 10 mL suspension aliquots were used for each dissolution profile. The sampling time points for tablets/capsules were 5, 10, 15, 30, 45, and 60 min, while those of suspensions were 10, 20, 30, 45, 60, and 90 min.

Dissolution samples at pH 1.2 were quenched with 0.2 M NaOH in order to stop further acidic degradation of clarithromycin. Suspension samples at pH 4.5 and 6.8 were filtered using 0.45 µm filters prior to HPLC analysis. Comparison of dissolution profiles was carried out by calculation of the similarity factor (f_2_) using Equation (1) [19].

(1) $$f_{2}=50\times \log\left\{{\left[1+\left(\frac{1}{n}\right)\sum \text{​}t=1^{n}{\left(R_{t}-T_{t}\right)}^{2}\right]}^{-0.5}\times 100\right\}.$$

*n*—number of testing time points, *R_t_*—average dissolution value of the reference product units at time t, and *T_t_*—average dissolution value of the test product units at time *t*.

## 3. Results and Discussion

### 3.1. Assay

All tablet/capsule samples complied with USP assay specifications, while all the suspensions tested complied with USP limits for pH [18] and assay. For purposes of interpretation, the USP (2014) assay limits for clarithromycin tablets (90.0%–110.0%) and suspensions (90.0%–115.0%) were applied as acceptance criteria. The assay results obtained are shown in Table 1.

The assay values for generic tablets/capsules formulations (CL1-CL11) were 98.4%–102.1%, while those of the innovator products Klaricid^®^ (CL15) and Klacid^®^ were 103.5% and 105.9% respectively. Suspension samples gave higher assay values (99.5%–110.0%) than tablets, with the innovator products Klacid^®^ and Klaricid^®^ (CL16) having the highest values at 109.6% and 110.1%, respectively.

### 3.2. Dissolution Profiles and Pharmaceutical Equivalence

#### 3.2.1. pH 1.2

**Figure 2:**
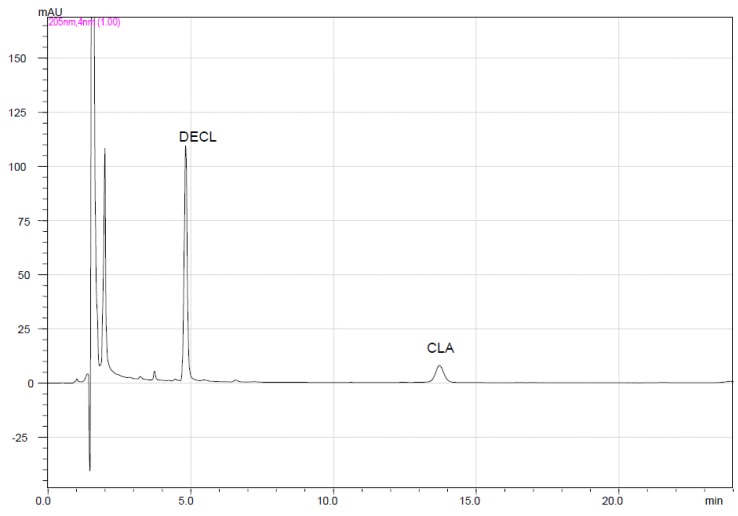
Typical chromatogram for clarithromycin dissolution (pH 1.2) obtained at 30 min. CLA—clarithromycin; DECL—decladinosyl clarithromycin. Chromatographic conditions: Column: XTerra^®^ C_18_ 5 μm (250 × 4.6 mm ID); column temperature: 56 °C; mobile phase: acetonitrile–0.2 M phosphate buffer, pH 6.80–water (40:3.5:56.5%, *v*/*v*/*v*); flow rate: 1.5 mL/min; detection: 205 nm; concentration: 0.43 mg/mL; injection volume: 100 µL.

Dissolution of clarithromycin from tablets/capsules at pH 1.2 proceeded with concomitant degradation to decladinosyl clarithromycin as the major product. A typical chromatogram of Klacid^®^ tablets at 30 min (Figure 2) shows good resolution between the clarithromycin peak and that of the major degrade decladinosyl clarithromycin.

The results for dissolution and degradation profiles for all samples tested at this pH are displayed in Table 2. In the table, the sum of clarithromycin and decladinosyl clarithromycin has been expressed as total clarithromycins. For this purpose, the areas of decladinosyl clarithromycin were corrected using normalization factors reported in the literature [8]. The similarity factors (f_2_) for each sample with respect to the innovator product Klacid^®^ tablets are also displayed in the table. Compliance for equivalence was set at (f_2_) ≥ 50 [19].

At this pH, low dissolution percentages were observed at early time points for some samples, leading to high variability and consequently a high percentage coefficient of variation values (CV, %). Out of the 13 tablet/capsule samples tested, five (CL1, CL3, CL10, CL11 and CL15) had CV, % <20 at 5, 10, and 15 min and CV, % <10 at 30, 45, and 60 min. Three samples (CL5, CL6 and CL8) had marginally raised CV, % values at a 5-min sampling time, CV, % <20 at 10 and 15 min, while CV, % at 30, 45 and 60 min ranged between 12.6 and 5.9. Notably, one capsule sample (CL4) had erratic dissolution with drug release occurring at once upon rapture of individual capsules, thus giving rise to CV, % >20 at all time points.

**Figure 3:**
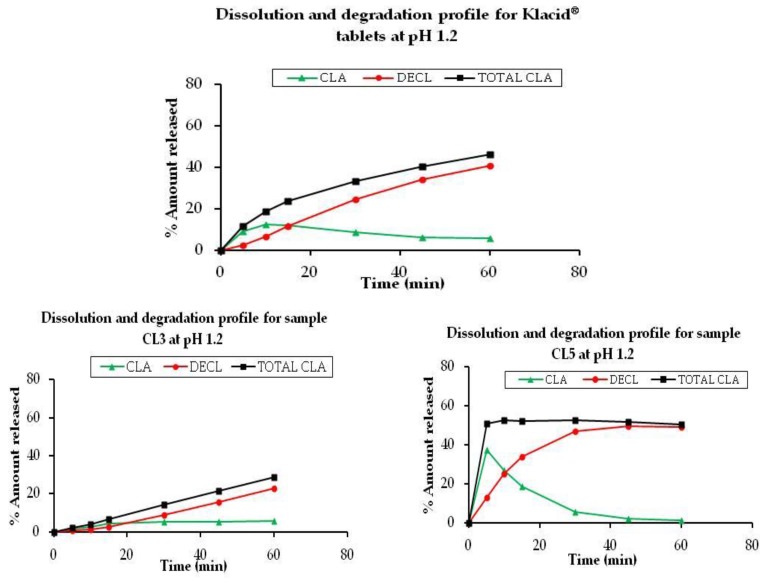

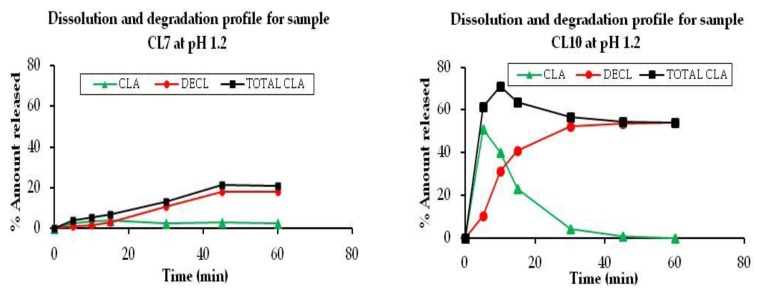
Dissolution and degradation profiles of Klacid^®^ tablets and samples CL3, CL5, CL7, and CL10 at pH 1.2.

Six tablet and capsule samples (CL1, CL4, CL6, CL8, CL11, and CL15) were found to be pharmaceutically equivalent to Klacid^®^, translating to a 50% success rate. Samples CL2, CL5, and CL10 were non-equivalent to Klacid^®^ on account of higher percentage total clarithromycins dissolved, while samples CL3, CL7, and CL9 were non-equivalent due to lower dissolution rates. Graphical presentations of the dissolution and degradation profiles for some non-equivalent samples are displayed in Figure 3 alongside that of Klacid^®^ tablets for comparison.

Klacid^®^ released 33% total clarithromycins within 30 min whereby the major component was decladinosyl clarithromycin (24%). Three generic products released over 45% total clarithromycins within 30 min. Samples CL5 and CL10 released 52% and 70% total clarithromycins, respectively, in the first 10 min while CL2 released 45% in 30 min. In both CL5 and CL10, clarithromycin degradation proceeded fast, producing 45%–50% decladinosyl clarithromycin within 30 min. Samples CL3, CL7, and CL9 had remarkably low dissolution values ranging 20%–29%, indicating significant formulation differences between them and the innovator product.

In all the four suspensions tested, no clarithromycin peak was detected, indicating that there was no drug release under this pH.

#### 3.2.2. pH 4.5

**Figure 4:**
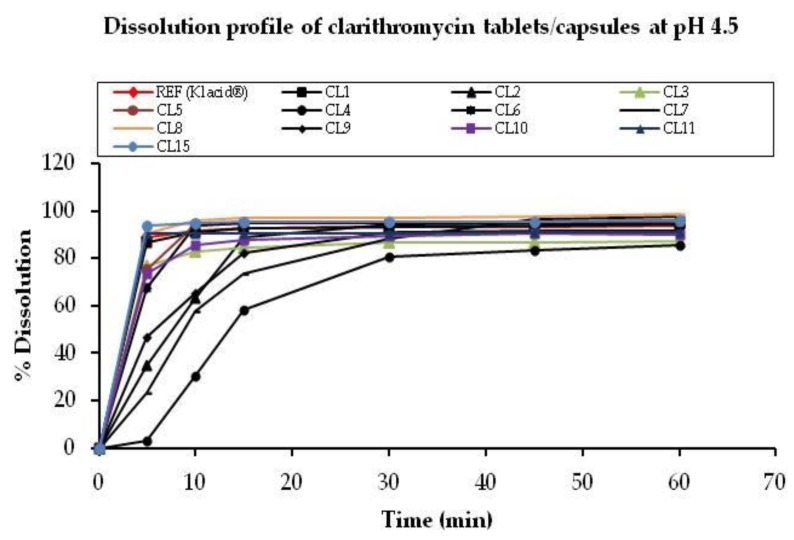
Comparative dissolution profile curves for clarithromycin tablets/capsules at pH 4.5. The CV, % values at this pH for 8 out of 13 samples tested (Klacid^®^, CL1, CL3, CL5, CL8, CL10, CL11, and CL15) were <20 at 5, 10, and 15 min sampling time and <10 at 30, 45, and 60 min. Sample CL6 had a CV, % of 20.9 at 5 min and <10 at the rest of sampling time points. Samples CL2, CL7, and CL9 had CV, % >20 at 5 and 10 min while the CV, % was <20 at 15 min and <10 at 30, 45 and 60 min. The capsule sample, CL4, yielded erratic dissolution with drug release occurring at once upon rapture of individual capsules, leading to CV, % >20 at the early sampling time points.

The average percentage dissolution of tablet/capsule samples at pH 4.5 were computed from peak areas, tabulated (Table 3), and plotted as a function of dissolution time (Figure 4). The similarity factors are as shown in Table 3.

All tablet/capsule samples released >80% of the drug within 30 min of dissolution. The highest release rate was 97% (CL8), while the lowest was 81% (CL4). No clarithromycin degradation was noted at this pH. Drug release for all suspensions at pH 4.5 was <2% within 90 min, probably due to enteric coating.

Samples CL2, CL4, CL7, and CL9 were found to be pharmaceutically non-equivalent to Klacid^®^. Non-equivalence in all these samples arose due to slow release rates at the early sampling points. The dissolution results indicate variability of product design, thus giving rise to differences in drug release characteristics despite the assay values being comparable to that of the innovator product.

#### 3.2.3. pH 6.8

**Figure 5:**
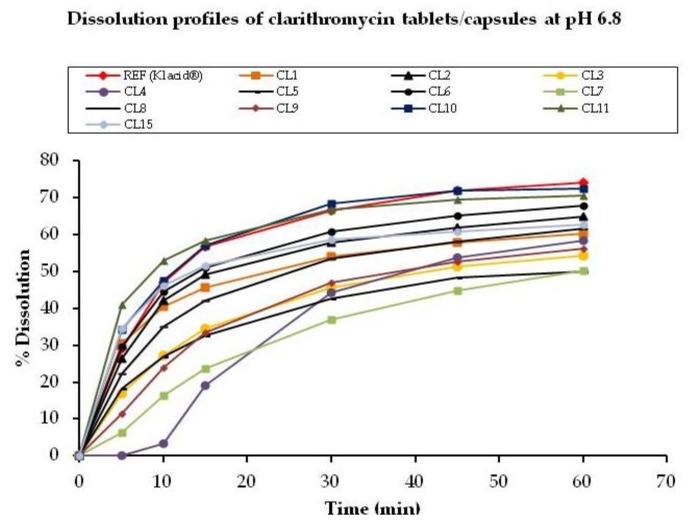
Comparative dissolution profiles of clarithromycin tablets/capsules at pH 6.8.

Enhanced dissolution was noted for all samples at pH 6.8 compared to pH 1.2 and pH 4.5. The percentage drug released at each time point as well as the similarity factors are recorded in Table 4 for tablets/capsules while those for suspensions are shown in Table 5. Comparative dissolution plots for tablet/capsule samples are as shown in Figure 5.

The CV, % values for nine out of the thirteen samples tested (CL1, CL2, CL3, CL6, CL7, CL8, CL10, CL11 and CL15) were <20 at 5, 10, 15 min and <10 at 30, 45 and 60 min. Klacid^®^ and CL9 had CV, % >20 at the two first sampling points and <10 at all other time points, while CL5 had CV, % <20 at 5, 10, and 15 min and a CV, % of 11.3–12.3 at 30, 45, and 60 min. The capsule sample, CL4, had CV, % >20 at the three early time points due to erratic drug release from individual capsule units.

Out of the 12 tablet/capsule products tested, 6 met the requirements for similarity factor (f_2_). Samples CL3, CL4, CL5, CL7, CL8, and CL9 were found to be non-equivalent to Klacid^®^ due to lower dissolution rates.

None of the four suspensions tested met the requirement for similarity factor (f_2_) relative to the innovator comparator product, Klacid^®^. The reference sample (Klacid^®^) and Sample CL16, being the innovator products by the same manufacturer, are expected to be equivalent. Their non-equivalence noted in the experiments carried out raises concerns about standardization of manufacturing sites as per the scale-up and post-approval changes (SUPAC) guidelines prescribed by the Centre for Drug Evaluation and Research (CDER) of the United States Food and Drug Administration (US FDA) [20]. Notably, Samples CL13 and CL14 yielded minimal drug release over the dissolution period, which raises valid concerns about their efficacy.

### 3.3. Comparative Evaluation for Similarity Factors

**Figure 6:**
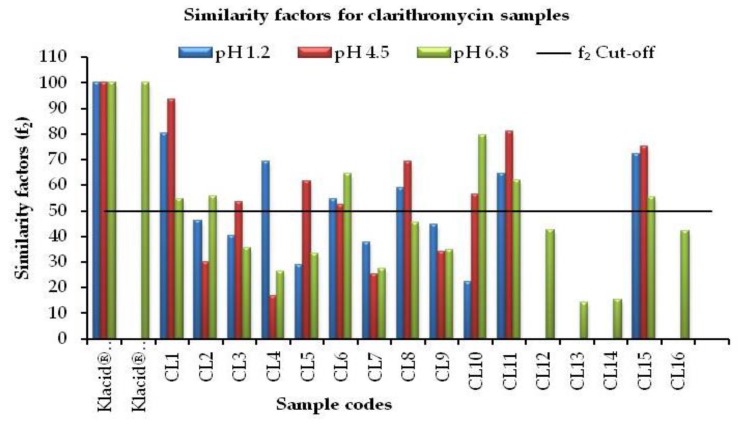
Graphical presentation of similarity factors for all clarithromycin samples tested at pH 1.2, 4.5, and 6.8.

A summary of f_2_ data for all analyzed samples is graphically illustrated in Figure 6. Overall, significant differences were observed in the dissolution profiles of the clarithromycin products tested. While all products complied with assay specifications, the majority of generic products did not comply with the specifications for similarity factor (f_2_) with the innovator products under the three-dissolution media used. Only 4 (25%) out of the 16 products tested met the acceptance criteria for similarity factor, (f_2_) relative to Klacid^®^ tablets and suspension. Notably, Samples CL7 and CL9 consistently failed to meet the f_2_ acceptance criteria at all three pH values. The results obtained indicate that the majority of clarithromycin generic products in the Kenyan market may not be pharmaceutically equivalent to the innovator products. Such products are deemed unacceptable according to the US FDA guidelines, which also recommend that further guidance be sought from the CDER to determine whether an in vivo study is appropriate for products with an f_2_ <50 [21].

## 4. Conclusions

In this study, it was noted that a significant percentage of clarithromycin generics did not comply with the specifications of similarity factor with respect to innovator products despite yielding comparable single-point dissolution results. At pH 1.2, sample CL2 yielded comparable results to the reference sample at 60 min run time, but failed to meet the specification for similarity factor. At pH 4.5, all 12 samples tested released ≥80% of the drug at a 30 min dissolution run time, which is comparable to pharmacopoeial specifications for a single point dissolution test, but four of these (CL2, CL4, CL7, and CL9) failed to meet the specifications for similarity factor with respect to the reference sample. Notably, Samples CL2 and CL9 had comparable results to the reference sample at 30, 45 and 60 min run time, while CL7 yielded comparable results at 45 and 60 min run time. The study findings may therefore point to the necessity of applying dissolution profile comparison with several dissolution time-points (five or six) as a measure of in vitro pharmaceutical equivalence between generic and innovator products, as single-point dissolution studies may not be sufficiently discriminative for the same.

The results obtained in this study will serve to inform regulatory authorities on the quality of clarithromycin products in the Kenyan market with respect to the pharmaceutical equivalence of generics to the innovator products. The findings indicate the need for extensive studies to establish pharmaceutical equivalence of generic products in the market for interchangeability.

## Figures and Tables

**Table 1:** Assay results of clarithromycin products analyzed (*n* = 9). Batch number omitted.

Sample Code	Dosage Form	Registration Status in Kenya	Assay as % Label Claim
Reference (Klacid^®^)	Tablet	R	105.9 (0.97)
Reference (Klacid^®^)	Suspension	R	109.6 (1.85)
CL1	Tablet	R	100.9 (0.87)
CL2	Tablet	R	100.2 (1.40)
CL3	Tablet	R	100.1 (0.56)
CL4	Capsule	R	102.1 (0.33)
CL5	Tablet	R	99.2 (0.39)
CL6	Tablet	R	99.3 (1.61)
CL7	Tablet	R	98.7 (0.38)
CL8	Tablet	R	101.5 (1.44)
CL9	Tablet	R	102.0 (1.13)
CL10	Tablet	R	98.8 (0.53)
CL11	Tablet	R	98.4 (0.39)
CL12	Suspension	R	107.8 (1.16)
CL13	Suspension	R	108.8 (0.88)
CL14	Suspension	R	99.5 (1.31)
CL15	Tablet	NR	103.5 (0.27)
CL16	Suspension	NR	110.1 (0.84)

R—Registered; NR—Not Registered. Figures in parentheses represent the coefficient of variation (*n* = 9 replicate injections).

**Table 2:** Dissolution and degradation profiles of clarithromycin tablets/capsules at pH 1.2.

Dissolution Time (min)	Average % Drug Released (*n* = 6)													
		Ref. (Klacid^®)^	CL1	CL2	CL3	CL4	CL5	CL6	CL7	CL8	CL9	CL10	CL11	CL15
5	CLA	8.97	8.41	14.08	1.43	5.16	37.64	2.29	2.61	3.77	0.88	53.90	5.39	11.70
	DECL	2.39	3.78	5.24	0.83	1.48	13.23	1.38	1.17	1.20	2.01	10.02	5.51	2.17
	TOTAL CLA	11.36	12.19	19.32	2.26	6.64	50.87	3.67	3.78	4.97	2.88	63.92	10.90	13.87
10	CLA	12.20	8.59	18.32	2.58	11.45	27.06	4.02	3.51	6.90	2.77	40.21	7.09	14.47
	DECL	6.48	8.94	11.43	1.16	5.37	25.42	3.60	1.63	4.60	5.03	30.46	5.00	4.18
	TOTAL CLA	18.67	17.53	29.75	3.74	16.82	52.48	7.62	5.14	11.50	7.80	70.67	12.09	18.65
15	CLA	11.89	7.58	23.16	4.25	11.88	18.48	5.60	3.78	7.96	2.43	20.99	7.93	17.33
	DECL	11.53	13.56	20.45	2.56	11.80	33.79	7.03	2.93	8.96	8.95	40.03	8.79	8.91
	TOTAL CLA	23.42	21.14	44.06	6.81	23.68	52.27	12.63	6.71	16.92	11.38	61.02	16.72	26.24
30	CLA	8.61	5.15	6.77	5.28	7.02	5.80	5.87	2.69	5.76	3.53	3.26	6.79	9.76
	DECL	24.50	25.17	38.53	8.97	23.00	46.82	18.24	10.57	20.25	18.78	51.48	20.06	20.77
	TOTAL CLA	33.12	30.32	45.31	14.25	30.02	52.62	24.12	13.26	26.01	22.31	54.74	26.85	30.53
45	CLA	6.10	3.83	1.81	5.45	2.99	2.14	5.37	3.16	3.84	2.11	0.48	5.67	6.04
	DECL	34.12	32.98	44.75	15.85	35.90	49.81	27.43	18.17	28.49	21.86	53.17	28.76	30.26
	TOTAL CLA	40.22	36.81	46.56	21.30	38.90	51.95	32.80	21.33	32.33	23.97	53.65	34.43	36.30
60	CLA	5.59	2.99	0.72	5.52	2.91	1.07	4.23	2.59	2.72	2.00	0.03	4.37	3.43
	DECL	40.44	38.21	43.39	23.04	33.90	49.35	40.40	18.20	35.44	25.45	54.10	36.56	35.27
	TOTAL CLA	46.04	41.20	44.11	28.55	36.81	50.42	44.63	20.79	38.16	27.45	54.13	40.93	38.70
**Similarity factor (f_2_)**		**100**	**80.4**	**46.0**	**40.2**	**69.2**	**28.8**	**54.6**	**37.9**	**58.9**	**44.8**	**22.2**	**64.7**	**72.2**

*n*—number of replicates; Ref.—reference product; CL1-CL15—sample codes; CLA—clarithromycin; DECL—decladinosyl clarithromycin; TOTAL CLA—total clarithromycin.

**Table 3:** Comparative dissolution profiles and f_2_ values for clarithromycin tablets/capsules at pH 4.5.

Time (min)	5	10	15	30	45	60	f_2_
Sample Code	% Clarithromycin Dissolved						
Ref (Klacid^®^)	88.82	91.60	92.92	93.12	93.31	93.68	**100**
CL1	86.91	91.23	92.54	93.20	93.81	94.48	**93.6**
CL2	34.56	63.32	88.80	93.10	94.78	94.87	**30.0**
CL3	77.06	82.74	84.27	86.46	86.86	87.24	**53.7**
CL4	2.93	30.43	58.14	80.76	83.44	85.34	**16.9**
CL5	75.45	94.19	94.86	95.25	95.37	95.64	**61.5**
CL6	67.53	93.94	94.61	94.99	95.39	95.77	**52.4**
CL7	23.40	57.86	73.47	88.46	96.52	97.75	**25.3**
CL8	90.62	95.85	96.88	97.27	97.40	98.44	**69.5**
CL9	46.87	65.30	82.15	91.31	91.79	91.42	**34.2**
CL10	73.71	85.81	87.73	89.57	90.59	90.16	**56.6**
CL11	90.57	90.67	90.64	90.66	90.88	90.91	**80.9**
CL15	93.65	94.87	95.17	95.22	95.56	95.78	**75.2**

**Table 4:** Comparative dissolution profiles and f_2_ factors for clarithromycin tablets and capsules at pH 6.8.

Time (min)	5	10	15	30	45	60	f_2_
Sample Code	% Clarithromycin Dissolved						
Ref (Klacid^®^)	29.02	46.97	56.71	66.41	71.87	74.02	**100**
CL1	30.34	40.55	45.64	54.16	57.86	60.14	**54.8**
CL2	26.43	42.11	49.13	57.82	62.02	64.75	**55.8**
CL3	16.99	27.18	34.56	45.51	51.30	54.19	**35.5**
CL4	0.04	3.39	19.16	44.35	53.73	58.36	**26.5**
CL5	18.19	27.02	32.74	42.57	48.25	50.03	**33.3**
CL6	29.50	44.49	51.00	60.70	65.19	67.89	**64.4**
CL7	6.35	16.22	23.58	37.02	44.92	50.22	**27.6**
CL8	22.32	34.95	42.07	53.53	58.23	61.50	**45.4**
CL9	11.47	23.80	33.44	46.96	52.65	56.17	**34.7**
CL10	34.18	47.53	56.96	68.28	71.99	72.43	**79.7**
CL11	40.94	52.83	58.27	66.66	69.44	70.66	**61.8**
CL15	34.44	46.21	51.73	58.53	60.87	62.75	**55.2**

**Table 5:** Dissolution profiles and f_2_ factors for clarithromycin suspensions at pH 6.8.

Time (min)	Percentage Drug Dissolved				
	Ref (Klacid^®^)	CL12	CL13	CL14	CL16
0	0	0.22	0.68	0	0.08
10	11.38	14.05	0.57	2.14	11.69
20	36.08	41.85	0.81	1.21	24.83
30	63.35	51.7	2.25	1.27	55.5
45	65.40	67.09	5.21	2.84	75.58
60	68.64	83.99	14.90	8.04	82.39
90	66.94	95.35	14.90	21.60	94.81
**Similarity factor (f_2_)**	**100**	**42.4**	**14.3**	**15.2**	**42.0**

## Declaration

This research was part of R.O.M.’s Master of Pharmacy thesis project (2014). The full thesis has been deposited in the University of Nairobi’s e-repository as per the University’s open access policy and is available at erepository.uonbi.ac.ke, while the PowerPoint presentation of the same is available at pharm-school.uonbi.ac.ke.
